# Prevalence and Risk Factors of Lungworm Infection in Small Ruminants in Selected Districts of Wolaita Zone, Southern Ethiopia

**DOI:** 10.1155/2024/6303598

**Published:** 2024-04-10

**Authors:** Wondimu Tessema, Minale Getachew, Ephrem Tora

**Affiliations:** ^1^Sodo Regional Veterinary Laboratory, Wolaita Sodo, Ethiopia; ^2^Arba Minch University, Arba Minch, Ethiopia

## Abstract

Among small ruminants, lungworms are important parasitic nematodes that infect the lower respiratory tract and are implicated in a high mortality and morbidity rate. However, the magnitude and its determinants of lungworm infection in the study districts are not known. The objective of this study was to estimate the prevalence of lungworm infection and assess associated risk factors in selected districts, Wolaita Zone, southern Ethiopia. A total of 742 fecal samples collected from 443 sheep and 299 goats were examined, using modified Baermann techniques, to recover first-stage larvae from fecal samples. Lungworms were detected in 271 (36.52%) samples infecting 192 (43.34%) sheep and 79 (26.42%) goats, respectively. Lungworm species Dictyocaulus filaria, Protostrongylus rufescens, and Muellerius capillaris were recovered in 114 (15.36%), 68 (9.16%), and 57 (7.68%) fecal samples, respectively. Mixed infection by two or more above species was noted in 32 (4.31%) of the samples. Among the determinants examined, agroecological environment, management system, and season showed statistically significant differences (*p* < 0.05) with the prevalence of infection in small ruminants. On the other hand, lungworm infection among sexes, age groups, and body condition scores showed no statistically significant difference (*p* > 0.05). The present study indicated that lungworm infection was a problem for sheep and goats in this study area. Thus, measures like deworming, early treatment, and improving animal husbandry should be practiced.

## 1. Background

Ethiopia has huge potential livestock populations and ranks first in Africa. The country has an estimated small ruminant population of around 31.30 million sheep and 32.74 million goats [1]. Small ruminants provide 33% of meat and 14% of milk consumption and account for 40% of cash income and 19% of the household meat consumption in the central highlands where mixed crop livestock production system is practiced [2]. Small ruminants are especially relevant in more extreme climates, and they are noted for their ability to convert low opportunity cost feed into high value products including meat, milk, fiber, manure, and hides [3]. While Ethiopia has a large population, its small ruminant farmers produce very little compared with other countries in sub-Saharan Africa [4]. This is because of disease, poor nutrition, poorly designed animal production systems, and inadequate veterinary services [5, 6].

Small ruminant lungworm parasites are extremely common, represent one of the biggest production bottlenecks, and reported in many tropical and subtropical locations around the world. This is because the tropical and subtropical environments are almost ideal for their survival and growth [5]. Among the species of worms involved in this type of infection with small ruminant are *Dictyocaulus filaria* (Rudolphi, 1809), *Protostrongylus rufescens* (Leuckart, 1865), and *Muellerius capillaris* (Müller, 1889), which are known to cause in the lower respiratory tract, a disease characterized by respiratory discomfort, called verminous bronchitis or verminous pneumonia or yet bronchopneumonia [7–9].

The pathogenicity of lungworms can be influenced by various factors, including the quantity of ingested infectious larvae, the robustness of the animals' immune system, and the location of the lungworms within the respiratory tract [7]. Regardless the pathogenicity, the clinical signs of infected animals with lungworm can be less obvious than signs of other livestock diseases [10]. Partly for this reason, such infections, besides those caused by gastrointestinal helminth parasites, are among the most neglected areas of veterinary care in much of the developing world, even considering that a high prevalence of lungworm infection is associated with poor production and economic losses [11].

A number of factors can affect lungworm infection in small ruminants. It has been recognized that lungworm infection in small ruminants is typically high, especially in the highland environments, of Ethiopia, characterized by intense rainfall and humidity [12]. These variations in the general prevalence in various regions could be attributed to variations in the animal's management practices, nutritional status, immunity level, presence of intermediate hosts, climate of the area, altitude, rainfall, humidity, temperature, and marshy areas for grazing, as well as sheep and goat management systems and examination season in their respective study areas [12, 13].

Despite the fact that the lungworm is a problem throughout Ethiopia, the majority of the available data are from the northern and western parts of the country. Limited information on the subject is available in the southern part of the country, the Wolaita Zone [14]. As Ethiopia is a country with diverse ecological and climatic conditions, occurrence, species diversity, and risk factors may vary from area to area. It is imperative to study this parasite in the current study area. The reports could be useful for animal owners and other organizations to develop control strategies. Thus, the objectives of this study were to estimate the overall prevalence of lungworm infection in small ruminants and to assess the associated risk factors.

## 2. Methods

### 2.1. Description of the Study Area

**Figure 1:**
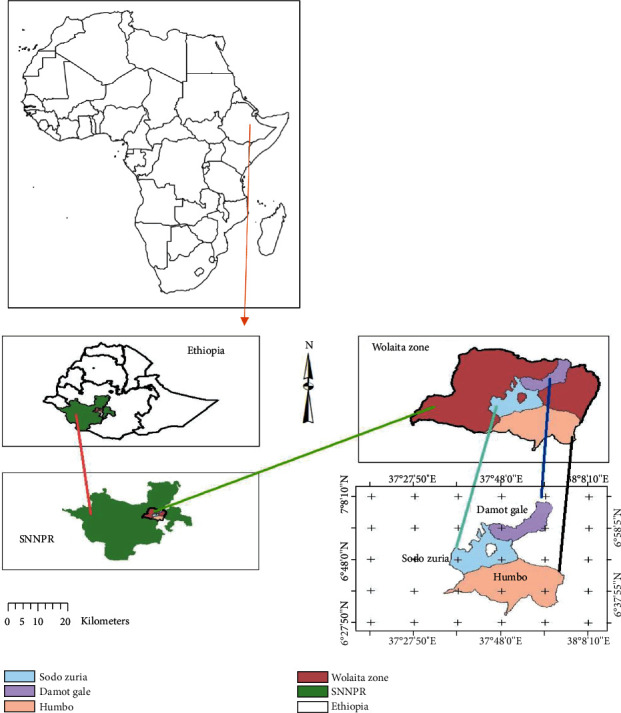
Map of Africa and Ethiopia displaying the locations of study areas.

The study was conducted in Sodo Zuria, Damot Gale, and Humbo districts in Wolaita Zone, SNNPRS, Ethiopia. These districts are located at a distance of 336 km, 318 km, and 356 km south of Addis Ababa between the coordinates of 6° 53′ 30^″^ and 7° 4′ 30^″^ N latitude and 37° 48′ 0^″^ and 37° 59′ 0^″^ E longitude, 6° 48′ 0^″^ and 6° 59′ 0^″^ N latitude and 37° 37′ 0^″^ and 37° 48′ 0^″^ E longitude, and 6° 30′ 0^″^ and 7° 0′ 0^″^ N latitude and 37° 30′ 0^″^ and 38° 10′ 0^″^ E longitude, respectively (Figure 1). The altitude of Sodo Zuria, Damot Gale, and Humbo districts is 1500 to 3200, 1500 to 2850, and 1001 to 2500 meters above sea level, and the average temperature varies between 15°C and 30°C, 12°C and 24°C, and 12.6°C and 20°C and average annual rainfall of 1201 to 1600 mm, 1001 to 1400 mm, and 1000 mm to 1200 mm, respectively. Sodo Zuria, Damot Gale, and Humbo districts have a livestock population of 234,120 cattle, 40,873 sheep, 13,833 goats, 118,289 poultry, 6087 donkeys, 58 horses, and 32 mules; 135,864 cattle, 39,758 sheep, 12,190 goats, 182,241 poultry, 16,128 donkeys, 1543 horses, and 1103 mules; and 173,569 cattle, 21,161 sheep, 42,739 goats, 197,532 poultry, 28,067 donkeys, 1159 horses, and 44,123 mules, respectively [15].

### 2.2. Study Population

In this study, sheep and goats were kept under extensive and semi-intensive management systems consisting of different ages, sexes, and body conditions and they were selected from 12 selected rural kebeles (Table 1).

### 2.3. Study Design

Cross-sectional study design was carried out from December 2019 to June 2020 to estimate the prevalence and associated risk factors of small ruminants' lungworm infection.

### 2.4. Sampling Techniques and Sample Size Determination

Multistage and stratified random sampling techniques were used to select the study area and sample of the study animals. Using multistage sampling technique, zone was selected as primary stage, districts as secondary stage, and kebeles as tertiary stage. Three districts, twelve kebeles, and four kebeles from each study district were purposely taken from Wolaita Zone based on agroecology and study animal population numbers.

Stratified random sampling was used to classify the study animals into strata. In the study, small ruminants were stratified into goats and sheep. After stratification, animals were sampled from respective kebeles using a simple random sampling technique. For this reason, the goat and sheep numbers in three districts were gathered from secondary data recorded in the livestock and fishery department of Wolaita Zone as well as respective districts and kebeles to reach the intended sample size. In Sodo Zuria, 13,833 goats and 40,873 sheep (a total of 54,706) were present; in Damot Gale, 12,190 goats and 39,758 sheep (a total of 51,948) were found, and in Humbo District, 42,739 goats and 21,161 sheep (a total of 63,900) small ruminant population was present [15]. Therefore, the sample size required for this study was determined depending on the expected prevalence of small ruminant lungworm infection and the desired absolute precision based on Thrusfield and Christley [16] by the following formula.

(1) $$\begin{array}{c} n=\frac{1.96^{2}\text{Pexp}\left(1-\text{Pexp}\right)}{d^{2}}, \end{array}$$

where *n* is the required sample size, Pexp id the expected prevalence, and *d* is the desired absolute precision (5%). Since there were published data at the national level by Asmare et al. [12] who reported 40.8% pooled estimates of lungworm infection in small ruminants in Ethiopia, the expected pooled prevalence for this study was 40.8%. Accordingly, the calculated sample size was 371. For increased precision, the sample size was doubled. Therefore, the total sample size required for this study was 742. After determining the total sample size for study animals in each study district and kebele, animals were proportionally allocated (Table 1).

### 2.5. Data Collection

#### 2.5.1. Animal Level Data Collection

During sampling in the field, age, sex, species, body condition score, agroecological environment, season, and management system of the animals were recorded in a preformed sample collection format. In Ethiopia, area from 2300 meters above sea level is highland, 1500–2300 is midland, and below 1500 meters above sea level is considered as lowland. Age of sheep and goats was determined by teeth according to the ESGPIP [17]. Those sheep and goats that have milk teeth that have started to wear down or are fully spread out were classified as young (less than or equal to one year) and those with erupted and growing 1st pair of permanent teeth were classified as adult (greater than and equal to one year). Body condition scoring was determined by differences in relative body fatness. Based on this body fatness, animals were categorized into three as poor, medium, and good, according to the ESGPIP [18].

#### 2.5.2. Parasitological Data Collection

Fecal samples were collected directly from the rectum of animals using a disposable plastic glove for the coprological examination. The fecal sample was put into screw-capped glass bottles and properly packed. All fecal samples were clearly labeled with the date of sampling, type of sample, and the identification number of the animal [19, 20]; then, the fecal sample was transported to the Sodo Regional Veterinary Laboratory for identification of the larvae (*L*_1_) of lungworms.

### 2.6. Parasitological Examination

The technique recommended by Urquhart et al. [19] was tasked to identify lungworm species from the collected samples. Using the modified Baermann method of detecting lungworm larvae in the laboratory, 5-10 g of fresh feces was weighed from each sample for the extraction of the first stage (*L*_1_) [21].

**Figure 2:**
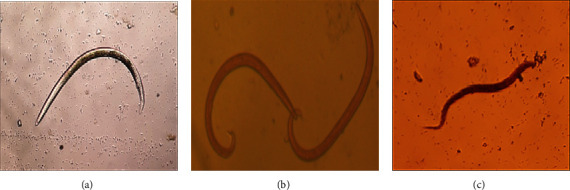
*L*_1_ larvae of lungworm identified during study period: (a) *Dictyocaulus filaria*; (b) *Muellerius capillaris*; (c) *Protostrongylus rufescens*.

Parasite larvae (*L*_1_) were identified by their morphological differences under a microscope. *D. filaria* larvae (*L*_1_) are distinguished from the other two lungworms by the characteristic cuticular knob on the anterior extremity, the large size and blunt tail, and intestinal inclusions on the larvae (*L*_1_), while the larvae of *P. rufescens* and *M. capillaris* are differentiated by their characteristic features at the tip of their tails. In both *P. rufescens* and *M. capillaris*, *L*_1_ is small and lacks an anterior cuticular knob. Further, *L*_1_ of *M. capillaris* has a dorsal spine at the pointed wave tail, but *L*_1_ of *P. rufescens* lacks a dorsal spine [22] (Figure 2).

### 2.7. Data Management and Analysis

The raw data and result of parasitological examination were entered and managed in MS Excel work sheet and analyzed by using STATA version 14.2. Descriptive statistics was performed to estimate the overall prevalence of the disease in small ruminants and the prevalence of parasitic infection by animal species, as well as the prevalence of each parasitic species. The prevalence of lungworm infection was calculated by dividing positive samples for the total number of samples examined. Binary logistic regression was employed to compute odds ratios (OR) and 95% confidence intervals (CI) in order to determine the strength of association between the statistically significant risk factors and parasite positivity. Considering a liberal *p* value (*p* = 0.25), predictors were checked for multicollinearity assessment using Goodman and Kruskal's gamma values. For multivariable regression modeling, backward stepwise selection method was set to determine predictors which were statistically considered as significant risk factors. A *p* value less than 0.05 at 95% confidence level was considered significant for all analyses. The model of multivariable logistic regression is as follows:

(2) $$\begin{array}{c} Y=\log\left(\frac{\pi_{i}}{1-\pi_{i}}\right)=\beta_{0}+\beta_{1}x_{1}+\beta_{2}x_{2}\cdots \cdots .+\beta_{n}x_{n}+\varepsilon_{n}, \end{array}$$

where *π*(*x*) = *P*(*Y* = 1|*X* = *x*) is a binary independent variable *Y* with two categories, *X* is a single predictor in the simple regression model, and *x*_1_, *x*_2_, ⋯, *x*_*n*_ are the predictors in the multivariable model.

## 3. Results

### 3.1. Overall Prevalence

A total of 742 (443 sheep and 299 goats) fecal samples were examined of which 271 were positive for lungworms with an overall prevalence of 36.52% (Tables 1 and 2). The prevalence of lungworms was much greater in sheep (43.34%) than goats (26.42%). Age-wise, the prevalence was 37.76% among young (<1 year old) and 35.47% among adults (>1 year old). Concerning sex, this study showed that a higher prevalence of lungworm infection was observed in females (38.67%) than males (32.84%). In the cases of body condition, the prevalence of lungworm infection was higher (39.60%) in animals of poor body condition than those of medium (37.46%) and good (30.23%). For the environments analyzed, prevalence of lungworms was higher in highland (45.37%) compared with midland (42.47%) and lowland (24.47%) (Table 2).

### 3.2. Proportions of Identified Lungworm Species

The proportion of different species was found to be different among positive fecal samples of small ruminants. It was found that 15.36% (114/742), 9.16% (68/742), 7.68% (57/742), and 4.31% (32/742) of the small ruminants were parasitized by *D. filaria*, *P. rufescens*, *M. capillaris*, and mixed infection of lungworms, respectively. *D. filaria* larva (*L*_1_) is the predominant species of lungworms detected through fecal samples from small ruminants (Table 3).

### 3.3. Univariable Logistic Regression Analysis of Associated Risk Factors

Univariable logistic regression analysis was used to determine single predictors having crude significance levels at *p* value < 0.25 that were of a prior interest for further multivariable logistic regression analysis by using the backward stepwise selection method (Table 4).

### 3.4. Multivariable Logistic Regression Analysis of Associated Risk Factors

During multivariable logistic regression analysis, backward stepwise regression analysis was used to select those predictors that predispose to the occurrence of lungworm infection in small ruminants in this study (Table 5). In the backward step-by-step regression analysis, the first step was selecting predictors with crude significance levels of *p* < 0.25 in the univariable logistic regression analysis. Finally, multivariable model was fitted containing predictors with adjusted significance levels at *p* value < 0.05.

The multivariable analysis for the lungworm infection in the different species of ruminants showed that there was a significant difference between sheep and goats (*p* < 0.05). It was observed that the odd of lungworm infection was 29.4% less likely in goats compared with sheep. In relation to the environments, the prevalence of lungworms varied significantly by altitude (*p* < 0.05). The odds of lungworm infection in the small ruminants in lowland areas revealed 70.4% less likely to be infected than those in highland areas. In contrast, the odds of lungworm infection in the small ruminants in midland area were 44.7% less likely to be infected than the ruminants in the highland area. The odds of lungworm infection in extensively managed small ruminants were 2.46 times higher than those semi-intensively managed ruminants. With regard to the seasonal distribution, there was a significant difference between wet and dry seasons (*p* < 0.05). Lungworm infection during the wet season (March, April, May, and June) was higher than along the dry season (December, January, and February) in the current study. The odd of lungworm infection was 26.4% less likely in dry season compared with wet season.

## 4. Discussion

This study proved that lungworm is one of the most widespread respiratory tract infections of small ruminants in the districts of Sodo Zuria, Damot Gale, and Humbo of the Wolaita Zone of the Southern Nations, Nationalities, and Peoples' Region, Ethiopia. Small ruminants could be infected with several species of lungworm [7, 19]. However, the most prominent respiratory disease-causing species identified in this study are *D. filaria*, *M. capillaris*, and *P. rufescens*. *D. filaria* is the predominant lungworm species infecting small ruminants, whereby causing poor body condition, clinical respiratory signs, and premature death [19, 22]. Thus, lungworm infections are serious health problems for small ruminant animals, which are likely to cause heavy economic losses.

The coproscopic study result showed an overall prevalence of lungworm infection of 36.5% in small ruminants in the study areas. The finding was almost in agreement with the reports of Addis et al. [23] (33.83% in Gonder town), Regassa et al. [24] (36.9% in Dessie and Kombolcha districts), Kassa et al. [25] (31.2% in three districts of South Wollo, Ethiopia), and Asmare et al. [12] (40.8% meta-analytic study in Ethiopia). Comparatively, this study documented lower prevalence than the reports of Alemu et al. [13], Abera et al. [26], and Fesseha and Mathewos [27] who reported overall prevalence of lungworm infection in small ruminants: 53.60%, 57.6%, and 44.02%, in Asella province, in Bale and Arsi zones, and in Durame District, southern Ethiopia, respectively. The results of this study, on the other hand, showed a relatively higher prevalence compared to reports of Asaye and Alemneh [28] (22.71% in Bahir Dar City, Amhara Region, Ethiopia) and Borji et al. [10] (10.85% in Mashhad of northeast Iran). The climate, altitude, intermediate hosts, favorable ecological conditions, the sheep and goat management systems of the respective study areas for the development of lungworm species, differences in the sample sizes used during the various investigations, seasonal variation during the investigation period, variation in the nutritional status of the small ruminants, and other factors may all contribute to the variation in the overall prevalence of lungworm infections in small ruminants across the study sites.

In the present study, the proportion of lungworm infection was higher in sheep (43.34%) than in goats (26.42%). This finding agrees with that of Regassa et al. [24] who reported 40.40% in sheep and 31.70% in goats in Dessie and Kombolcha districts, northeastern Ethiopia, and Kadi et al. [29] who also reported 57.40% in sheep and 31.20% in goats from Asella, Arsi Zone, southeast Ethiopia. This finding contradicts that of Alemu et al. [13] who reported a higher proportion of goats (50.70%) than sheep (24.46%) in northeastern Ethiopia and Tenaw and Jemberu [30] who also reported a higher percentage of goats (36.3%) than sheep (15.5%). The possible explanation for the difference among the two species of small ruminants might arise from the differences in grazing behavior of the two species of animals. The fact that sheep are predominantly grazers means that they have a greater chance of ingesting large numbers of infective larvae (*L*_3_) than goats. Since goats usually browse, they are less likely to ingest infective larvae.

The current study found that 37.76% of lungworm cases were in young animals and 35.47% in adults, which were comparatively similar and had no statistically significant differences (Table 3). This finding aligns with Asaye and Alemneh [28], who documented relatively higher prevalence of lungworm infection in young animals (36.5% against 29.5% in adults) in and around Bahir Dar City, Amhara Region, Ethiopia. Negash et al. [31] also observe a similar pattern of infection by lungworm, when reported higher prevalence in young animals (53.3%) than he did in adults (36.3%) in Gedeb Asasa District, West Arsi Zone, Ethiopia. Furthermore, the present finding does not corroborate the results reported by Regassa et al. [24] who documented 22.5% of young animals infected and 77.5% of adults, in Dessie and Kombolcha districts, northeastern Ethiopia. Though statistically not significant, the smaller difference observed in the present results could be owing the animal's vulnerability to lungworm infection which declines with age, and young animals are more likely to have the infection than adults. This indicates that due to previous exposure to lungworm infection, mature animals have developed immunity. Because young animals are exposed less frequently than older animals, young animals are much more vulnerable [32]. Comparative similarity could be due to the proportion of young and adult animals sampled in this study since the study was conducted outdoors, where more adult animals were sampled.

The predominant parasitic species in the study area was *D. filaria* followed by *P. rufescens*. This finding is supported by Alemu et al. [13] and López and Martinson [7] who found *D. filaria* to be more prevalent than other species. In advance, Asmare et al. [12] confirmed the predominance of *D. filaria* by systematic review and meta-analysis in Ethiopia. The possible explanation for the prevalence of *D. filaria* in the study area might be related with the life cycles of these parasites. Thus, *D. filaria* has a direct life cycle and requires shorter time to develop to an infective stage. According to Hansen and Perry [11] and López and Martinson [7] after ingestion, the larvae of this parasite could be shed with feces within 5 weeks. In contrast to *D. filaria*, *P. rufescens* and *M. capillaris* exhibit epidemiologically complex transmission patterns including the host, the parasite, and an intermediate host.

In this study, the odds of lungworm infection was 70.6% higher in sheep than in goats. This finding is in line with the report by Regassa et al. [24] who stated an odd of lungworm infection was 2 times higher in sheep compared with goats in Dessie and Kombolcha districts, northeastern Ethiopia. However, it disagrees with previous reports provided by Tenaw and Jemberu [30], who indicated higher odds of lungworm infection (3 : 2–5; *p* < 0.001) in goats than sheep, and Alemu et al. [13] and Borji et al. [10], who stated that goats are more susceptible than sheep. Goats are more susceptible to *D. filaria*, according to a controlled experimental investigation [33]. Despite this susceptibility being demonstrated during experiments, they are naturally browsers in their feeding habits. As a result, their risk of contracting lungworms is probably lower than that of sheep, which typically graze and are therefore anticipated to have a lower incidence of lungworms. This study claims that the reason this was not detected was because goats were compelled to graze due to a lack of browsing forages in the study location, which may have increased exposure to lungworms.

With regard to the environment, small ruminants in lowland areas were 70.4% less likely to be infected by lungworm than the small ruminants kept in highland areas. This is in agreement with Alemu et al. [13], who reported lower odds of prevalence for *D. filaria* and *M. capillaris* at low altitudes when compared to high altitudes in northeastern Ethiopia. Asmare et al. [12] and Yimer and Desie [34] showed different prevalence among various types of agroecological environments in northern Ethiopia. Environmental conditions play a large role in determining the geographical distribution and prevalence of lungworm infection. This might be due to the effect of altitude which is attributable to climatic parameters. That is, the survival and development of lungworm larvae are favored by low moisture content and high humidity. Based on this, in highlands, due to moisture, lungworm species survive better than in lowland agroecology.

With regard to management systems, the occurrence of lungworm infection was significantly different. The odds of lungworm infection in extensively managed small ruminants were 2.46 times higher than those in semi-intensively managed ruminants. This result is consistent with that of Abebe et al. [14]. Small ruminants kept in extensive and semi-intensive management systems showed a statistically significant difference, according to a study by Alemu et al. [13]. In this case, animals managed under extensive management systems presented a higher chance of contracting lungworm infection than animals managed under semi-intensive management systems, because animals under extensive management systems repeatedly graze on the pasture, which increases the chances of contracting lungworms, but in cases of semi-intensive management, animals have a low chance of pasture contamination, which leads to a low risk of lungworm infection. Animals kept under semi-intensive management practices were fed balanced, palatable, and nutritious feed, which increased their immunity against lungworm infections, as opposed to those kept under intensive management, which did not get enough feed, which compromised their immunity and allowed the parasites to grow and infect the animals continuously for a long period of time. In contrast, animals kept in extensively managed systems do not feed adequately, which could compromise their resistance and expose them to parasite larvae on a constant basis.

This study found that lungworm infections are significantly different during the wet season and the dry season, based on the seasonal distribution of lungworm infections. Lungworm infection in the wet season was higher than in the dry season in this study area. The odds of lungworm infection were 26.4% less likely in the dry season compared with the wet season. This finding coincides with previous reports by Regassa et al. [24]. This could imply that the epidemiology of lungworms indicates that a damp and cool environment is very suitable for the development of *D. filaria* and the third stage larva (*L*_3_) is resistant to cold. The dung beetles facilitate the spread of *D. filaria* larvae under favorable conditions, but under dry conditions, the larvae may be inhibited in the lung. Moisture is a vital factor in determining the survival and availability of parasites [35]. Thus, relatively higher records of lungworm infection during the wet season could also be due to the fact that the survival and development of larvae are favored by low moisture and high humidity.

## 5. Conclusions

Lungworm infection in small ruminants is highly prevalent and widely distributed in the study districts, Sodo Zuria, Damot Gale, and Humbo of the Wolaita Zone, and significant variation was noted between the two host species. Hence, lungworm infections can be serious health problems for small ruminant animals, which are likely to cause heavy economic losses. The lungworms species recorded in this study were *D. filaria*, *P. rufescens*, and *M. capillaris*, and the most common infection was caused by *D. filaria*, though mixed infections were also observed. Statistical tests were used to analyze a number of risk factors affecting prevalence, including species, age groups, sexes, body condition, agroecology, management method, and season. The prevalence of lungworm infection shows statistically significant association with risk factors such as species, agroecology, management system, and season. There was no statistically significant variation in the prevalence of lungworm infection among sexes, age groups, and physical conditions. Hence, the prevention and management of lungworm infections should be taken into account. To enable the creation of a useful control strategy in the study area, further research on seasonal risk variables is required.

## Figures and Tables

**Table 1:** Population of small ruminants and their proportional allocation in sample in the study districts.

District	Kebele	Study population	Proportionately sampled animals
Sodo Zuria	Waraza Lasho	3470	58
	Dalbo Wogene	2542	43
	Kuto Sorphela	5043	88
	Dalbo Atwaro	3176	49

Damot Gale	Shasha Gale	2486	47
	Gacheno	3086	57
	Harto Kontolo	3174	57
	Harto Burkuto	3586	60

Humbo	Ampo Koysha	2778	54
	Ela Kebela	3438	66
	Ambe Shoya	4518	87
	Koysha Wongela	3700	71

Total			742

**Table 2:** Prevalence of lungworm infection and associated risk factors in the study areas.

Risk factors	No. of examined	No. of positive	Prevalence	95% CI
Species				
Sheep	443	192	43.34%	38.2–47.4
Goat	299	79	26.42%	21.4–29.8
Age				
Adult	464	166	35.47%	29.8–39.4
Young	278	105	37.76%	33.8–40.4
Sex				
Male	274	90	32.84%	28.7–34.9
Female	468	181	38.67%	33.8–42.4
Body condition score				
Good	172	52	30.23%	27.3–33.7
Medium	315	118	37.46%	33.8–42.4
Poor	255	101	39.60%	36.8–41.4
Agroecological environment				
Highland (Sodo Zuria)	238	108	45.37%	43.8–48.4
Midland (Damot Gale)	226	96	42.47%	41.2–44.6
Lowland (Humbo)	278	67	24.47%	22.5–27.4
Management system				
Semi-intensive system	236	67	28.38%	21.4–29.8
Extensive system	506	204	40.31%	38.8–42.3
Season				
Wet season	302	131	43.37%	40.4–46.7
Dry season	440	140	31.81%	28.4–4.2
Total	742	271	36.52%	33.4–39.5

CI: confidence interval.

**Table 3:** Prevalence and identified lungworm species of small ruminants (*n* = 742) in study area.

Species of lungworm	Worms in sheep	Worms in goat	No. of positive	Prevalence	95% CI
*Dictyocaulus filaria*	83	31	114	15.4%	10.2–19.8
*Protostrongylus rufescens*	50	18	68	9.2%	6.8–14.2
*Muellerius capillaris*	44	13	57	7.7%	4.3–11.5
Mixed infection	21	11	32	4.3%	1.8–7.3
*D. filaria and P. rufescens*	13	6	19	2.5%	1.2–4.1
*D. filaria and M. capillaris*	8	5	13	1.7%	0.8–3.4
Total	198	73	271	36.5%	29.8–42.4

CI: confidence interval.

**Table 4:** Univariable logistic regression analysis of associated risk factors in the study areas.

Risk factors	Coefficients	Crude OR	*p* value	95% CI
Species				
Sheep	Ref.			
Goat	-0.669	0.734	0.000	0.369-0.710
Age group				
Adult	Ref.			
Young	-0.104	0.901	0.508	0.663-1.225
Sex				
Male	Ref.			
Female	-0.249	0.778	0.115	0.570-1.063
Body condition				
Good	Ref.			
Medium	-0.110	0.895	0.524	0.638-1.257
Poor	-0.431	0.649	0.039	0.431-0.978
Agroecology				
Highland	Ref.			
Midland	-0.583	0.533	0.002	0.387-0.802
Lowland	-1.276	0.889	0.000	0.183-0.425
Management system				
Semi-intensive system	Ref.			
Extensive system	0.833	2.300	0.000	1.664-3.180
Season				
Wet season	Ref.			
Dry season	-0.333	0.716	0.030	0.529-0.968

Ref: reference category.

**Table 5:** Multivariable logistic regression analysis of associated risk factors.

Risk factors	Coefficients	Adjusted OR	*p* value	95% CI
Species				
Sheep	Ref.			
Goat	-0.570	0.706	0.001	0.4000–0.798
Sex				
Male	Ref.			
Female	-0.249	0.778	0.115	0.570-1.063
Body condition				
Good	Ref.			
Medium	-0.110	0.895	0.524	0.638-1.257
Poor	-0.431	0.649	0.039	0.431-0.978
Agroecology				
Highland	Ref.			
Midland	-0.591	0.553	0.000	0.380–0.805
Lowland	-1.216	0.296	0.002	0.191-0.458
Management system				
Semi-intensive system	Ref.			
Extensive system	0.902	2.465	0.000	1.759-3.453
Season				
Wet season	Ref.			
Dry season	-0.305	0.736	0.030	0.335–0.912

Ref: reference category.

## Data Availability

The entire required data are available and recorded in the format of text we have attached as supporting information files (Supplementary data.Txt).

## Abbreviations

CI: Confidence interval
CSA: Central statistical authority
L: Larvae
OR: Odds ratio
SNNPR: Southern Nations, Nationalities, and Peoples' Region.
